# Measuring Health-related Quality of Life in Pediatric Patients with Ultra-rare Diseases: A Multicenter Study

**DOI:** 10.2174/0109298673363460250603053204

**Published:** 2025-06-23

**Authors:** Osama Y. Muthaffar, Mahmoud A. Gaddoury, Mohammed A. Alsubaie, Anas S. Alyazidi, Sarah Y. Bahowarth, Maha K. Alghamdi, Ahmed K. Bamaga, Muhammad Imran Naseer

**Affiliations:** 1 Department of Pediatrics, Faculty of Medicine, King Abdulaziz University, Jeddah, Saudi Arabia;; 2 Healthcare Strategy Department, NEOM, Tabuk, Saudi Arabia;; 3 Faculty of Medicine, King Abdulaziz University, Jeddah, Saudi Arabia;; 4 Department of Internal Medicine, King Faisal Specialist Hospital & Research Center, Jeddah, Saudi Arabia;; 5 Institute of Genomic Medicine Sciences (IGMS), King Abdulaziz University, 21589, Jeddah, Saudi Arabia

**Keywords:** Clinical genetics, rare diseases, ultra-rare diseases, epilepsy, DNA, pathogenic variation

## Abstract

**Background:**

Ultra-rare diseases (URDs) are defined based on point prevalence and are classified as conditions affecting fewer than 1 in 50,000 individuals, and they are more likely to exist among communities with higher consanguinity rates requiring evidence-based data.

**Methods:**

In this multi-center study, we used next-generation sequencing to identify 30 pediatric patients with URDs. Along with the demographic information about their parents, clinical, laboratory, and radiological data was also obtained. Multinomial regression was carried out to assess statistical differences and determine associations using the Quality of Life of Childhood Epilepsy (QOLCE)-55 scale.

**Results:**

There were 19 male (63.33%) and 11 (36.67%) female patients. Their current age range was 2-15 years (mean=8.83 years). The majority were diagnosed with sodium channelopathy (64.51%). The average Quality of Life (QoL) score of all participants was 51.43 ± 9.01 (reference range 0-100) with quartiles Q1=40, Q2=43.5, and Q3=56.

**Conclusion:**

We propose that URDs complicated by epilepsy can significantly impair the QoL of patients and their families.

## 1 INTRODUCTION

Extremely rare diseases, also termed ultra-rare diseases (URDs), are pathogenic conditions with a prevalence of less than one case per 50,000 individuals [1]. This is in comparison to rare diseases that are regarded to affect roughly less than one case per 1650 individuals in the United States [2] and less than one case per 2000 individuals in Europe [3]. With no general definition of rare and URDs, the legislative framework is closely related to a definition based on point prevalence [4, 5]. Despite their rarity, the cumulative global frequency of known rare diseases can be as high as 6%, affecting around 450 million people worldwide [5, 6]. They are considered an emerging public health priority that requires evidence-based data to inform public policies [5]. In the Middle East, there are insufficient, reliable data to understand the prevalence and epidemiology of rare diseases and URDs. It is, however, expected that the Middle East region will bear higher rates of such diseases due to its Mendelian mode of inheritance, which surges in a region of prevalent relative marriages and large family structures [7]. It is also crucial to remember that recent advances in genome testing have transformed the detection of rare diseases [8]. In recent years, healthcare planners have recognized that measuring disease alone is not a sufficient indicator of health status. Instead, they have discovered that measuring the quality of life (QoL) using validated tools provides more insight into the attention, learning, physical pain, and health-related quality of life (HRQoL) associated with chronic illnesses [9, 10]. Among rare diseases are chronic conditions, including epilepsy, a medical condition characterized by a predisposition to generate abnormal electrical activity in the brain. This activity manifests as seizures and is accompanied by neurobiological, cognitive, psychological, and social consequences. Epilepsy affects patients of all ages, races, social classes, and geographical regions [11-13]. Despite its brief duration, a seizure activity creates a post-seizure impairment with intermittent disruption of normal body function with episodes of loss of consciousness and injuries in which its recurrence poses a challenge to the patient's QoL [14]. Patients with epilepsy, especially children, further experience social stigma and misconceptions that deteriorate their overall health and disease progress [10]. Moreover, it affects them economically, socially, and culturally, including the education of young children and adolescents [15-17]. Moreover, families of such chronically ill patients were reported also to have a number of physical health difficulties [18, 19]. To address the aforementioned gaps and scarcity of data, we collected detailed data on a group of patients diagnosed with a URD complicated by chronic epileptic activity, some of whom were diagnosed using advanced genetic testing in a highly-populated and diverse city on Saudi Arabia's western coasts, as well as the QoL of their caregiving family members.

## 2 MATERIALS AND METHODS

### 2.1 Study Design and Setting

Following the Strengthening of the Reporting of Observational Studies in Epidemiology (STROBE) checklist for retrospective studies [20], we conducted a multi-center retrospective cohort study on 30 children diagnosed with URDs. The diagnostic criteria for a URD include the following components: disease prevalence of 1:50,000 or lower and the disease registration in the National Organization for Rare Disorders (NORD) database for rare diseases. This criteria was approved in agreement with the National Institute of Clinical Excellence's (NICE) definition of URD [21].

### 2.2 Study Procedure

Using the pediatric neurology outpatient electronic hospital record, we extracted data on all pediatric visits from three tertiary care centers in Jeddah, Saudi Arabia, for the previous 12 months. The time duration of the sample collection was between 16-30 September 2023. The first center is a publicly funded and owned educational tertiary care center that serves the entire community with 750 beds. The second facility is a privately operated tertiary care center with a bed capacity of 300. The third facility is a large national referral center that focuses on challenging situations for patients with special requirements and offers advanced medical services with 500 beds. These centers were chosen based on the accessibility of their data, as well as to ensure broad representation and variety. Data was extracted directly from the electronic record to minimize data entering errors. The retrieved variables were sorted into two categories. In the first aspect, data were based on the affected pediatric patients, including their genetic background (*i.e.*, genetic variant, homozygosity, alleles, genetic test used for diagnosis), demographic data (gender, ethnicity, origin, current age, age of diagnosis, type of admission (emergency, out-patient), baseline data (laboratory workup), hospital outcome, birth parameter (term, weight, height, head circumference, pregnancy outcome, and neonatal period), and seizure information. In the second aspect, data were focused on the primary caregivers of the affected patients. The extracted variables included their age, gender, household income, parental status, and QoL assessment. Household income was classified into upper class, upper middle class, middle class, lower middle class, and lower class based on their self-reported income.

### 2.3 Instruments

We assessed QoL using a pre-validated tool termed the Quality of Life in Childhood Epilepsy (QOLCE-55) questionnaire version 1.0 [22]. It is a scale created to compare baseline risk factors that predict HRQoL in children newly diagnosed with epilepsy. It employs the standard test theory approaches, where scores are considered to be a linear relationship of their true score and error, and the error variation between persons is assumed to be constant [23]. Its items are assessed on a five-point Likert scale, with Cronbach's alpha ranging from 0.82 to 0.97 for individual subscales and 0.96 for the overall measure. The instrument's convergent and divergent validity was verified by correlations with the Child Health Questionnaire-Parent Form [24]. We contacted parent using phone calls and applied the instrument after clarifying the study's purposes and research objective and obtaining their written consent at their next clinic visit. The scale was adopted in its original form without modifications. It consisted of four scales, namely, cognitive, emotional, social, and physical. The scale has eleven sub-scales designed to assess various dimensions, including anxiety, depression, self-esteem, memory, behavior, language, attention/concentration, social activities, social interaction, physical constraints, and other aspects of cognition. The instrument's coding (*i.e.*, QOLCE-55 scale) was conducted following the guidelines provided with the questionnaire. During the patients’ interview, we adopted an Arabic version of the questionnaire that was validated and developed by Khalil *et al.* [25]. During the pilot phase, the instrument demonstrated high test-retest reliability, with intra-class correlation coefficients for all questionnaire domains ranging from 0.91 to 0.98. The results of Cronbach's alpha denoted good internal consistency. Convergent and divergent validity checks revealed that items from all domains had a significant correlation with their scale scores. We employed the translated version of the instrument on the same population that was used in the pilot study, namely the caretakers of children with epilepsy, to ensure the appropriate assessment for our cohort.

### 2.4 Sample Collection

Next-generation sequencing (NGS) was used to identify patients with URDs. In order to carry out treatments, we acquired the genetic samples of the patients at King Abdulaziz University Hospital (KAUH) and adhered to the proper ethical and logistical precautions implemented in the local institution. To confirm the pathogenic variation, whole exome sequencing (WES) testing was performed on the patients. Using an Illumina array and NGS-based copy number variation (CNV) analysis, deoxyribonucleic acid (DNA) capture probes were used. The gene's coding regions and both coding and non-coding variations that are known to be pathogenic or expected to be so were the focus of the analysis. Software that has been validated was used for data processing, variant calling, and annotation [26]. The Online Mendelian Inheritance in Man database (OMIM) was used to further prioritize rare variants that were homozygous missense, affecting disease-associated genes, or were predicted to cause loss of function (frameshift, nonsense, and splice site mutations). To predict the pathogenicity of the discovered variation, a number of prediction methods were employed. With the exception of benign or probably benign variants, all variations associated with the patient's phenotype were determined. Furthermore, clinical data and family history were used to assess all the variations.

### 2.5 Statistical Analysis

Data were collected in a Microsoft Excel (Microsoft^®^ Corp., Redmond, WA, USA) version 20. Mean scores in scales and subscales of QOLCE-55 were calculated. The means and standard deviations of numerical variables were examined and described. Quantitative variables were described using measures of central tendency. To examine statistical differences and determine a correlation between the children's sociodemographic and clinical traits and their quality of life, one-way analysis of variance (ANOVA) and multinomial regression were carried out. Fisher's exact test and the Likelihood Ratio (LR) chi-square statistic were conducted to determine variable independence. The chi-squared test was employed to compare nominal parameters. The data was post hoc tested using the Bonferroni correction. As previously mentioned in the literature, the sample size was not predetermined because there were more than twelve subjects [27]. In our sampling technique, we adopted the formulation of Julius (2005), which suggests a minimum sample size of 12 subjects per treatment arm [27]. A *p*-value of <0.05 and 95% confidence intervals were considered statistically significant. Statistical analysis was performed using IBM SPSS Statistics for Windows, version 27 (IBM Corp., Armonk, NY, USA).

## 3 RESULTS

### 3.1 Sociodemographic Characteristics of the Patients

Thirty pediatric patients with a diagnosis of URDs characterized by seizures were included; 11 (36.7%) were females, and 19 (63.3%) were males. The geographical distribution revealed a predominant representation of patients from the western (40%) and southern (26.7%) regions of Saudi Arabia. A significant portion (53.3%) of the patients fell within the school-aged category. *SCN1A* mutation was identified in 19 (63.3%) patients, and additional mutations included GAMT, *PRF1*, *SZT2*, *PCDH19*, *ROGDI*, *ATP1A3*, and *SHANK3*. The majority of households (73.3%) were upper-middle class in terms of socioeconomic standing. Additionally, 80% of the children did not have health insurance.

### 3.2 Quality of Life in Patients with Ultra-Rare Diseases

Patients diagnosed with URDs reported an overall mean QoL score of 51.43 ± 9.01. Fig. (**1**) presents a visual representation of the mean scores for each subscale, indicating that patients exhibited the highest QoL in terms of physical functioning (61.99 ± 14.89). Conversely, the lowest QoL was observed in relation to cognitive functioning (42.36 ± 19.96). A descriptive analysis was carried out to examine the relationship between sociodemographic characteristics and QoL scores in a subgroup comprising 19 participants with the *SCN1A* variant (Table **1**). Results showed no statistically significant differences in QoL scores based on gender, origin, age group, socioeconomic status, or health insurance coverage. Additionally, a linear regression analysis was performed on a subgroup with the *SCN1A* variant to examine the association between socioeconomic factors and QoL scores (Table **2**). While several factors were investigated, none demonstrated a statistically significant relationship with QoL. Gender and health insurance showed positive but non-significant associations, whereas household income and age exhibited negative but non-significant associations. These findings suggest that socioeconomic factors may not be major predictors of QoL within this specific subgroup.

### 3.3 Impact of Sociodemographic Factors on Quality of Life

Table **3** depicts the sociodemographic parameters and their association with QoL in URD patients. Male patients had a slightly higher average (52.07 ± 9.05) than their female counterparts (50.32 ± 9.27). Moreover, patients hailing from the central region of Saudi Arabia reported the highest QoL (62.60 ± 1.96) compared to individuals from other regions. School-aged children demonstrated the lowest QoL (49.80 ± 8.40) among different age groups. Among the genetic mutations, the highest QoL was observed in a patient with *SHANK3* mutation (61.19), closely followed by a patient with *ATP1A3* mutation (57.10). Conversely, patients with *ROGDI* and *PCDH19* mutations reported the lowest QoL (48.94 and 48.89, respectively). Families classified as upper class in socioeconomic status exhibited the lowest QoL (48.19 ± 7.02). Children with health insurance demonstrated a comparatively better QoL (53.30 ± 6.54) than those without health insurance (50.97 ± 9.59). Despite these changes, none of the sociodemographic factors revealed a statistically significant difference in the mean total QoL. A linear regression analysis examined the association between socioeconomic factors and QoL scores. While several factors were investigated, none demonstrated a statistically significant relationship with QoL (Table **4**). Gender, genetic variant, and health insurance showed positive but non-significant associations, whereas household income, age group, and origin exhibited negative but non-significant associations. These findings suggest that socioeconomic factors may not be major predictors of QoL.

### 3.4 Impact of Clinical Factors on Quality of Life

A complete assessment of the clinical parameters and their association with the QoL in patients with URDs is presented in Table **5**. Regarding seizure types, individuals experiencing focal seizures reported the highest QoL (60.76 ± 5.17), followed by those with atonic seizures (58.72 ± 7.44) and myoclonic seizures (58.08 ± 4.39). Generalized tonic-clonic seizures, the most prevalent seizure type, were associated with an average QoL of 48.99 ± 7.52. Patients with status epilepticus and complex partial seizures had the lowest QoL (40.65 and 40.00, respectively). Additionally, individuals with their first epileptic seizures occurring between 6 months and 11 months of age exhibited the lowest mean QoL score (48.28 ± 10.54).

### 3.5 Impact of Antiseizure Medications Response on Quality of Life

When patients were categorized based on their ASM response and compared for QoL, those with a response less than 50% exhibited a mean QoL of 49.27 ± 9.49. Patients with a response between 50% and 79% demonstrated a mean QoL of 50.93 ± 8.32, while those with a response of 80% or higher had a significantly higher mean QoL of 57.59 ± 5.32. Fig. (**2**) illustrates the relationship between antiseizure medication response and QoL in patients with URDs. Notably, an increase in ASM response was associated with a better QoL, with patients reaching a response of 80% or more showing a statistically significant higher overall mean QoL score (*p* = 0.010).

## 4 DISCUSSION

Evaluating the QoL in pediatric patients with URDs is integral to providing comprehensive care. Given that many URDs lack specialized or widely available treatments due to their rarity and that focused therapies may be limited or unavailable, it helps healthcare providers focus on improving general well-being and mitigating symptoms. Additionally, it provides counseling and support services to pediatric patients and their relatives who are living with URDs, which might lead to psychological effects. Publishing such research contributes to understanding the disease's impact and potential areas for improvement and guiding future research efforts. It also supports advocacy initiatives by providing evidence of the unique needs of these patients.

A prominent but varying characteristic of many URDs is epilepsy, often stemming from complex genetic mutations that cause a variety of neurological and systemic symptoms [28]. The heterogeneity of these diseases is highlighted by the fact that epilepsy is important in conditions like *GNAO1*-related neurodevelopmental disorders, but it may be overshadowed by other symptoms like movement disorders or developmental delays [29]. The genetic basis of epilepsy in these populations is highlighted by the frequent association of ultra-rare de novo genetic variants with developmental and epileptic encephalopathies (DEE), a severe epilepsy phenotype [30, 31]. However, the relative importance of epilepsy's symptoms varies greatly depending on the particular ailment; some diseases, like Lennox-Gastaut and Dravet syndromes, are characterized by epilepsy, while others place more emphasis on non-epileptic characteristics [32-34]. We reported an overall mean QoL score of 51.43 ± 9.01 for the included studied patients diagnosed with URDs characterized by seizures. This is consistent with what has been investigated in a previous study that was conducted to assess the QoL among pediatrics with intractable epilepsy by Marghalani *et al.*, where the reported mean ± SD of overall QoL was 43.02 ± 15.70 [35]. In our study, we investigated various sociodemographic characteristics, including factors, such as gender, origin, age group, genetic variant, socioeconomic status, and health insurance. Surprisingly, our analysis revealed no statistically significant differences in the mean total QoL scores across these factors (*p*-value >0.05). These findings are consistent with those previously noted by George *et al.*, who found no meaningful correlation between the demographic factors and the total Quality of Life in Epilepsy Inventory-10 scores [36]. However, upon further investigation into the nature of seizures, we established a significant statistical association between the response to antiseizure medications, specifically achieving a response rate of ≥ 80%, and the overall QoL (*p*-value = 0.010). In a study on ASMs and QoL in individuals with epilepsy, Dwivedi *et al.* discovered that the respondents' group had a considerably better overall quality of life (*p* <0.0001) than the non-responders' group [37]. Their QoL scores were in line with earlier research by Thomas *et al.*, which found that patients who are responding well to ASM typically have higher QoL than those who are not in a number of ways, such as stable daily activities due to fewer seizures and improved safety and social interactions [38]. It also enables the patient's independence and alleviates emotional and medical burdens. Our findings suggest that the nuanced complexity associated with QoL in URDs complicated with chronic illnesses should be explored in more depth in different types of epilepsies and other neurological disorders. Addressing specific barriers to limited QoL will be important to the long-term outcome of children.

## CONCLUSION

In conclusion, the most evident result of the study is that treatment response is the primary factor influencing quality of life. Hence, providing appropriate ASM will significantly improve the QoL in those patients. The response to ASM showed a higher effect on the QoL than any other socio-demographic variables, as also reported in previous literature on the importance of clinical improvement in comparison to other variables. Patients with URDs and a coexisting chronic condition, such as epilepsy, experienced similar determinants of QoL as those with chronic disease alone and no identifiable genetic etiology.

## LIMITATIONS OF THIS STUDY

The study could be potentially limited by the recall bias of the participants. Moreover, we were unable to conduct certain statistical tests due to the sample size.

## Figures and Tables

**Fig. (1):**
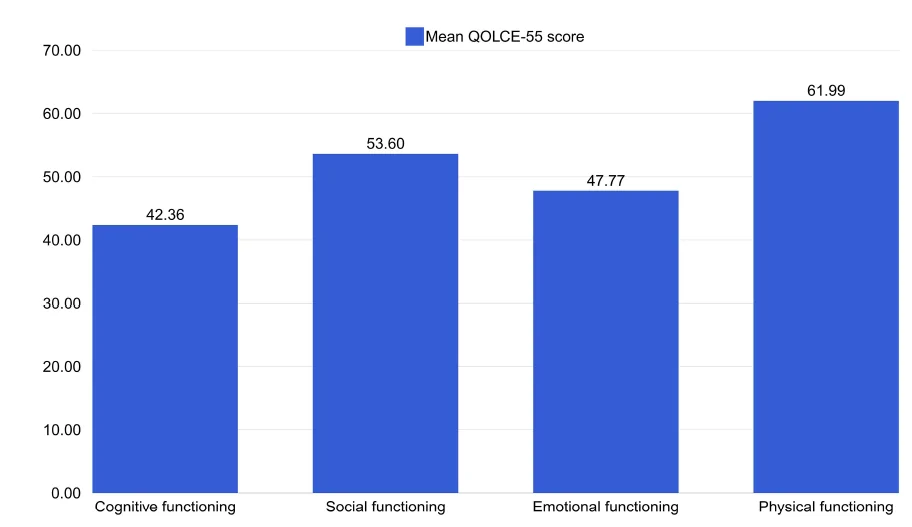
Mean scores of all sub-scales of the “QOLCE-55” scale in URD patients.

**Fig. (2):**
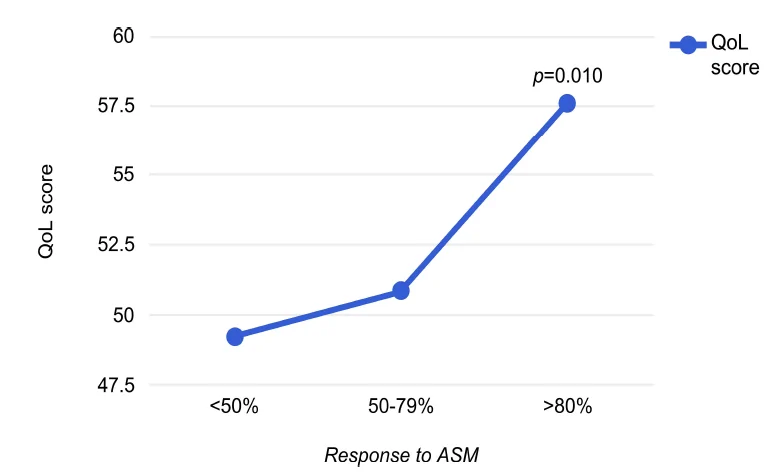
Relationship between antiseizure medication response and QoL in URD patients. *p-value is considered statistically significant at *p* < 0.05.

**Table 1:** Sociodemographic characteristics and their relationship with the QoL in *SCN1A* variant subgroup.

**Characteristic**	**Number (%)**	**Overall Mean QOL Score (SD)**	***p-Value***	***t-Value***	***F-Value***
**Gender**					
Male	7 (36.8)	47.14 (9.72)	0.333	-0.997	0.004
Female	12 (63.2)	51.79 (9.84)			
**Origin**					
West Saudi	6 (31.6)	49.70 (10.08)	0.897	-	0.312
South Saudi	8 (42.1)	61.21 (0.00)			
North Saudi	2 (10.5)	45.79 (12.53)			
Central Saudi	1 (5.3)	49.16 (11.62)			
East Saudi	1 (5.3)	53.46 (0.00)			
South-east Saudi	1 (5.3)	52.68 (0.00)			
**Age Group**					
Preschool	5 (26.3)	49.19 (9.77)	0.577	-	0.569
School age	10 (52.6)	48.63 (9.19)			
Adolescent	4 (21.1)	54.81 (12.53)			
**Socioeconomic Status**					
Upper class	1 (5.3)	53.16 (0.00)	0.460	-	0.816
Upper middle class	17 (89.5)	49.21 (9.90)			
Lower middle class	1 (5.3)	61.80 (0.00)			
**Health Insurance**					
Yes	2 (10.5)	54.74 (2.23)	0.111	-0.702	7.639
No	17 (89.5)	49.53 (10.23)			

**Table 2:** Linear regression analysis of socioeconomic factors predicting QoL score for *SCN1A* variant subgroup.

**Predictor**	**B**	**Std. Error**	**Standardized β**	**t-Value**	**p-Value**	**95% Confidence Interval for B**	
						**Lower Bound**	**Upper Bound**
Gender	4.665	5.300	.236	.880	.395	-6.784	16.114
Origin	.480	1.525	.088	.315	.758	-2.815	3.775
Age Group	1.368	4.248	.098	.322	.752	-7.810	10.546
Household Income	-5.589	5.928	-.299	-.943	.363	-18.396	7.217
Health Insurance for Participant	8.632	9.367	.278	.922	.374	-11.604	28.869

**Table 3:** Sociodemographic characteristics and their relationship with the QoL in URD patients.

**Characteristic**	**Number (%)**	**Overall Mean QOL Score (SD)**	***p-Value***
**Gender**			
Male	19 (63.3)	53.11 (8.72)	0.905
Female	11 (36.7)	48.85 (8.85)	
**Origin**			
West Saudi	12 (40)	49.18 (9.13)	0.505
South Saudi	8 (26.7)	52.17 (9.78)	
North Saudi	5 (16.7)	49.29 (7.90)	
Central Saudi	2 (6.7)	62.60 (1.96)	
East Saudi	1 (3.3)	53.46	
South-east Saudi	1 (3.3)	52.68	
Pakistani	1 (3.3)	61.19	
**Age Group**			
Toddler	2 (6.7)	52.77 (6.12)	0 .946
Preschool	7 (23.3)	50.91 (10.58)	
School age	16 (53.3)	51.03 (8.32)	
Adolescent	5 (16.7)	53.62 (11.17)	
**Genetic Variant**			
SCN1A	19 (63.3)	50.26 (9.60)	0.945
GAMT	3 (10)	54.06 (10.40)	
PRF1	2 (6.7)	53.65 (12.67)	
SZT2	2 (6.7)	52.97 (6.40)	
PCDH19	1 (3.3)	48.89	
ROGDI	1 (3.3)	48.94	
ATP1A3	1 (3.3)	57.10	
SHANK3	1 (3.3)	61.19	
**Socioeconomic Status**			
Upper class	2 (6.7)	48.19 (7.02)	0 .612
Upper middle class	22 (73.3)	50.73 (9.27)	
Middle class	4 (13.3)	55.86 (8.15)	
Lower middle class	1 (3.3)	61.80	
Lower class	1 (3.3)	48.89	
**Health Insurance**			
Yes	6 (20)	53.30 (6.54)	0.116
No	24 (80)	51.11(9.42)	

**Abbreviations: **QoL, quality of life; SD, standard deviation. A *p*-value is considered statistically significant at *p* < 0.05. Test of significance: ANOVA and unpaired t-test.

**Table 4:** Linear regression analysis of socioeconomic factors predicting QoL score. B: Unstandardized coefficient indicates the direction and magnitude of the relationship. Standardized β reflects the strength of the effect in standardized units. The t-value and *p*-value indicate the significance of each predictor (*p*<0.05 suggests statistical significance). A 95% Confidence Interval (CI) indicates the range of plausible values for BBB; intervals including zero suggest non-significant effects.

**Predictor**	**B**	**Std. Error**	**Standardized β**	**t-Value**	***p*-Value**	**95% Confidence Interval for B**	
						**Lower Bound**	**Upper Bound**
Gender	2.263	3.806	0.123	0.595	0.558	-5.610	10.137
Origin	-0.238	1.213	-0.042	-0.196	0.846	-2.746	2.270
Age Group	-0.390	2.620	-0.035	-0.149	0.883	-5.811	5.030
Genetic Variant	0.818	1.047	0.193	0.781	0.443	-1.348	2.983
Household Income	-2.422	2.818	-0.208	-0.860	0.399	-8.251	3.407
Health Insurance for Participant	2.134	4.994	0.096	0.427	0.673	-8.198	12.466

**Table 5:** Clinical characteristics and their relationship with the QoL in URD patients.

**Characteristic**	**Number (%)**	**Overall Mean QOL Score (SD)**	***p-Value***
**Type of Seizures**			
Generalized tonic-clonic seizure	23 (76.7)	47.64 (6.78)	0.219
Febrile seizures	12 (40)	51.50 (10.52)	
Myoclonic seizures	7 (23.3)	58.08 (4.39)	
Focal seizures	4 (13.3)	60.76 (5.17)	
Atonic seizures	4 (13.3)	58.72 (7.44)	
Tonic seizure	3 (10)	57.37 (5.80)	
Nocturnal generalized tonic-clonic seizure	1 (3.3)	40.65	
Complex Partial Seizures	1 (3.3)	59.81	
Status Epilepticus	1 (3.3)	40.65	
**Age at First Epileptic Seizure**			
< 6m	5 (16.7)	54.96 (4.67)	0.557
6m-11m	11 (36.7)	48.61 (10.27)	
1y-3y	12 (40)	52.47 (8.54)	
> 3y	2 (6.7)	53.65 (12.67)	
**Response to Antiseizure Medications**			
≥ 80% response	7 ()	57.59 (5.33)	0.010*
< 80% response	23 ()	49.56 (9.15)	
**Motor and Tone Abnormalities**			
Infantile hypotonia	4 (13.3)	56.05 (5.40)	0.082
Abnormal gait	6 (20)	52.79 (9.82)	0.437
Ataxia	2 (6.7)	52.94 (11.66)	0.836
**Behavioral and Developmental Abnormalities**			
Autism Spectrum Disorder	3 (10)	55.71 (6.56)	0.257
Attention-Deficit / Hyperactivity Disorder	7 (23.3)	49.96 (10.19)	0.560
Global Developmental Delay	8 (26.7)	52.04 (9.22)	0.889
**Radioelectrographical Abnormalities**			
Abnormal EEG	16 (53.3)	51.34 (9.13)	0.517
Abnormal MRI	5 (16.7)	52.80 (9.95)	0.315

**Abbreviations: ** QoL, quality of life; SD, standard deviation. *p-value is considered statistically significant at *p* < 0.05. Test of significance: ANOVA and unpaired t-test.

## Data Availability

The datasets generated and/or analyzed during the current study are not publicly available to protect patients' privacy. However, deidentified data can be made available from the corresponding author on reasonable request and with the appropriate IRB approvals (https://www.springernature.com/gp/authors/research-data-policy/data-availability-statements).

## LIST OF ABBREVIATIONS

URDs: Ultra-rare Diseases
QoL: Quality of Life
NGS: Next-generation Sequencing
WES: Whole Exome Sequencing
CNV: Copy Number Variation
DNA: Deoxyribonucleic Acid

## STANDARDS OF REPORTING

STROBE guidelines were followed.
